# Effects of photoperiod on nutrient digestibility, hair follicle activity and cashmere quality in Inner Mongolia white cashmere goats

**DOI:** 10.5713/ajas.18.0154

**Published:** 2018-08-27

**Authors:** Chong Zhi Zhang, Hai Zhou Sun, Sheng Li Li, Dan Sang, Chun Hua Zhang, Lu Jin, Marco Antonini, Cun Fa Zhao

**Affiliations:** 1 Institute for Animal Nutrition and Feed, Inner Mongolia Academy of Agricultural and Animal Husbandry Sciences, Hohhot, 010031, China; 2 Italian National Agency for new Technology, Energy and Sustainable Economic Development, ENEA UTAGRI Inn CR Casaccia, Roma, Italy

**Keywords:** Cashmere Goat, Digestibility, Melatonin, Photoperiod, Secondary Hair Follicle

## Abstract

**Objective:**

This study investigated the effects of photoperiod on nutrient digestibility, hair follicle (HF) activity and cashmere quality in Inner Mongolia white cashmere goats.

**Methods:**

Twenty-four female (non-pregnant) Inner Mongolia white cashmere goats aged 1 to 1.5 years old with similar live weights (mean, 20.36±2.63 kg) were randomly allocated into two groups: a natural daily photoperiod group (NDPP group:10 to 16 h light, n = 12) and a short daily photoperiod group (SDPP group: 7 h light:17 h dark, n = 12). All the goats were housed in individual pens and fed the same diets from May 15 to October 15, 2015. The digestibility of crude protein (CP), dry matter (DM), and neutral detergent fiber (NDF) were measured in different months, along with secondary hair follicle (SHF) activity, concentration of melatonin (MEL), and cashmere quality.

**Results:**

Although there was no significant difference in the live weights of goats between the SDPP and NDPP groups (p>0.05), the CP digestibility of goats in the SDPP group was significantly increased compared to the NDPP group in July, September, and October (p<0.05). For the DM and NDF digestibility of goats, a significant increase (p<0.05) was found during in September in the SDPP group. Furthermore, compared to the NDPP group, the SHF activity in July, the MEL concentration in July, and the cashmere fiber length and fiber weight in October were significantly increased in the SDPP group (p<0.05).

**Conclusion:**

The cashmere production of Inner Mongolia white cashmere goats was increased without obvious deleterious effects on the cashmere fibers in the SDPP group (metabolizable energy, 8.34 MJ/kg; CP, 11.16%; short daily photoperiod, 7 h light:17 h dark).

## INTRODUCTION

As a dynamic mini-organ, the skin hair follicle (HF) undergoes periodic transformations throughout its lifetime. The HFs in cashmere goats are divided into primary hair follicles (PHFs) and secondary hair follicles (SHFs); the cashmere with economic value is produced by SHFs, which are different from PHFs in both morphogenesis and function [1]. The growth progress of cashmere in goats is affected by photoperiod, nutrition, management, and environmental and genetic factors [2–6]. The cashmere exhibits a seasonal pattern arising from circannual changes in the natural photoperiod, and the number, function, and activity of the SHFs control the cashmere growth [7,8]. The skin HFs undergo recurrent cycling of controlling anagens, catagens, and telogens with a defined periodicity; short duration of sunshine accelerates the development of HFs, while the longer duration of sunshine retards the development of HFs, ultimately halting their development.

The light conditions associated with seasonal changes can regulate the endocrine and nervous systems of animals. The pelage development of many fur-bearing animals is related to the changes in photoperiod and is controlled by a variety of hormones including melatonin (MEL) [9–12]. The cashmere grows most vigorously in the autumn, which has a photoperiod. The secretion of MEL in cashmere goats shows distinct cyclical changes with photoperiod, and this cycle is consistent with the growth cycle of cashmere. Studies have shown that MEL is a critical intermediary between photoperiod and cashmere growth, and the greater level of MEL secreted by the pineal gland during times of short daily photoperiod may be the key factor for cashmere growth via increasing the activity of SHFs [11,13–15]. Previous studies have shown that the photoperiod is associated with mammary development consistent with the enhancement of milk yield in the ensuing lactation [16–18]. Photoperiod regulates the HF growth from telogen to anagen and modifies circadian rhythm gene expression in HFs [19], and improves body weight, meat quality of breast muscle in broilers [20]. Furthermore, recent studies indicate that not only the yield, diameter (diam.), length of the cashmere fiber, regularity of methane (CH_4_), carbon dioxide (CO_2_), and ammonia (NH_3_) gases concentration are changed significantly [14,21], but also the HFs can be induced to enter the full anagen phase early [5] by activating these periodic key regulators and extending the SHFs full-anagen phase via a short daily photoperiod [6].

Inner Mongolia white cashmere goats are economically im portant for their cashmere, which is hailed as a “soft gold” by the textile industry. Moreover, the seasonal rhythm of cashmere growth is well known to be related to photoperiod. However, the mechanism of how a short photoperiod affects cashmere growth remains unknown. Therefore, in order to explore the mechanism of cashmere growth underlying HF cycling and the fiber growth potential in cashmere goats, we investigated the effects of photoperiod on nutrient digestibility, HF activity, and cashmere quality in Inner Mongolia white cashmere goats.

## MATERIALS AND METHODS

### Ethics statement

In order to minimize goats’ suffering, all skin tissue samples were collected under local procaine hydrochloride anesthesia according to the International Guiding Principles for Biomedical Research Involving Animals. The Animal Ethics Committee of the Inner Mongolia Academy of Agriculture and Animal Husbandry Sciences (Hohhot, China), which is responsible for Animal Care and Use in the Inner Mongolia Autonomous Region of China, approved the experimental protocols used in this study (approval number IMAAAHS# 1215000046002373XP) [5].

### Experiment time and place

The experiment was performed at the animal husbandry science and technology farm in Otog Front Banner, Inner Mongolia, China (latitude 38° 23′N, longitude 108° 07′E, altitude 1,378 m). The experiment was conducted from May 15 to October 15, 2015, and the entire experimental period was 150 days.

### Animal, feeding and photoperiod management

Twenty-four female (non-pregnant) Inner Mongolia white cashmere goats aged 1 to 1.5 years old with similar live weights (mean, 20.36±2.63 kg) were randomly allocated into two groups: a natural daily photoperiod group (NDPP group:10 to 16 h light, n = 12) and a short daily photoperiod group (SDPP group: 7 h light:17 h dark, n = 12). The diet of the goats in the two different groups was the same and consisted of a mixture of concentrate and roughage at a ratio of 40%:60%. The nutrition level of the goat basal diet was referenced from the National Research Council (NRC) [22]. The goats were provided with feed containing 1.2 times the metabolizable energy (ME) and 11.16% crude protein (CP). Each group had twelve goats. The feed composition of the NDPP and SDPP groups is listed in Table 1 (dry matter [DM] basis).

Following two weeks’ acclimatization, the formal experiment was conducted from May 15 to October 15. All of the goats were housed in individual pens and fed diets twice a day at 9:00 am and 15:00 pm, and the goats had free access to water and a mineral mixture block. The diet and remaining feed of each goat were weighed in order to determine the goat’s dry matter intake (DMI).

**Figure 1:**
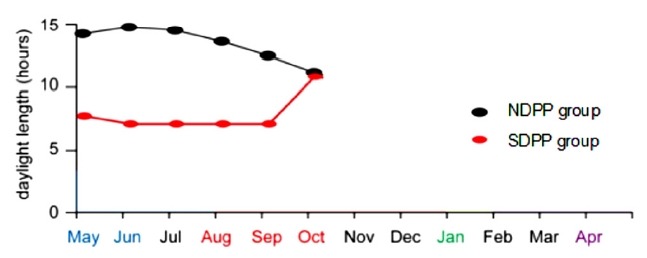
Daylight lengths of different months in the NDPP and SDPP groups^1)^. The goats in the SDPP group were housed in a dark shed (there was no light in the shed, and ventilators were used to circulate air) with less than 0.1 lux of opacity and good air conditions from 16:00 pm to 9:00 am daily (exposing the goats to 17 h of dark and 7 h of light (17 D:7 L). The goats in the NDPP group were housed outside the dark shed and were exposed to the natural photoperiod (exposing the goats to 10 to 16 h of light). The SDPP group’s shortened photoperiod treatment ran from May 15 to October 15, 2015. ^1)^ Natural daily photoperiod group (NDPP group: 10 to 16 h light, n = 12), short daily photoperiod group (SDPP group: 7 h light:17 h dark, n = 12).

The goats in the SDPP group were housed in a dark shed (there was no light in the shed, and ventilators were used to circulate air) with less than 0.1 lux of opacity and good air conditions from 16:00 pm to 9:00 am daily (exposing the goats to 17 h of dark and 7 h of light (17 dark:7 light). The goats in the NDPP group were housed outside the dark shed and were exposed to the natural photoperiod (exposing the goats to 10 to 16 h of light) (Figure 1). The SDPP group’s shortened photoperiod treatment ran from May 15 to October 15, 2015.

### Collection of feces and assays of dry matter, crude protein, and neutral detergent fiber digestibility

All of the experimental goats were used for digestibility measurements, and the feces from five days were collected using feces bags and dried at 105°C. Samples were obtained in July, September, and October. The feces were collected and weighed twice a day before the goats were fed their diets.

The DM, CP, and neutral detergent fiber (NDF) digestibility (GB/T6435-1986, GB/T6432-94, GB/T5009-2003) were determined by the total feces collection method using the following formula[23]:

Apparent digestibility=100-[feces weight×nutrient content in feces/DMI×nutrient content in the diet (%)]

### Collection of skin and assay of secondary hair follicle activity

**Figure 2:**
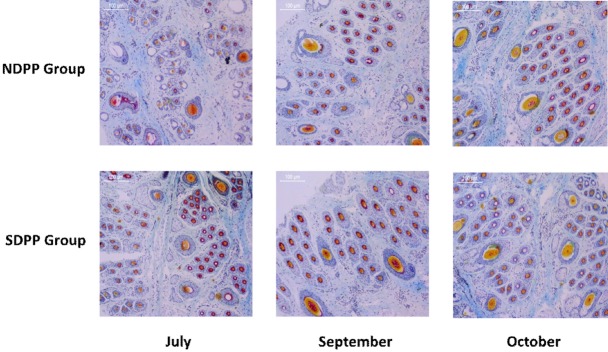
Effects of photoperiod on secondary hair follicle activity (magnification 100×) of goats in July, September, and October^1)^. The horizontal axis means diferent months and the vertical axis means different groups. Skin samples from the edge of the scapula of all of the experimental goats were collected using a 1 cm diameter ring drill during July, September, and October. First, the skin tissue samples were rinsed with phosphate-buffered saline (pH = 7.4) and immediately placed in paraformaldehyde fixative solution (0.1 *M*, pH = 7.4). After 48 h, the skin tissue samples were dehydrated using an alcohol gradient, paraffin-embedded, and continuously sectioned at 6 μm. Then, the tissues were dyed by using the sapic method and subsequently examined using a Nikon microscope. Finally, each section had five images taken, and each specimen was observed under a Nikon microscope and an Instudio camera system. The total number of secondary hair follicles in 10 random fields was counted (magnification 100×), and the percentage of secondary hair follicle activity was calculated [7]. ^1)^ Natural daily photoperiod group (NDPP group: 10 to 16 h light, n = 12), short daily photoperiod group (SDPP group: 7 h light:17 h dark, n = 12).

Skin samples from the edge of the scapula of all of the experimental goats were collected using a 1 cm diam. ring drill during July, September, and October. First, the skin tissue samples were rinsed with phosphate-buffered saline (pH = 7.4) and immediately placed in paraformaldehyde fixative solution (0.1 *M*, pH = 7.4). After 48 h, the skin tissue samples were dehydrated using an alcohol gradient, paraffin-embedded, and continuously sectioned at 6 μm. Then, the tissues were dyed by using the sapic method and subsequently examined using a Nikon microscope. Finally, each section had five images taken, and each specimen was observed under a Nikon microscope and an Instudio camera system. The total number of SHFs in 10 random fields was counted (magnification 100×), and the percentage of SHF activity was calculated [7], as SHF activity = active SHFs/total SHFs×100% (Figure 2).

### Assay of melatonin concentration in plasma

Jugular vein blood samples (10 mL) from all of the experimental goats were obtained at 8:00 am before feeding during July, September, and October. The blood was collected into heparinized tubes and centrifuged (3,500 g, 15 min), and the serum was stored at −20°C. The MEL concentration was determined by ELISA (20161226, Fankeshiye Corp., Shanghai, China) [14].

### Detection of cashmere fiber diam, length, weight, and net fiber rate

Cashmere and wool samples from behind the right shoulder blade were dyed on May 15 and were shorn near to the skin on October 15. Both dyeing and shearing samples measured 10×10 cm. The cashmere fibers were separated from the wool, was washed, dried, and weighed [13]. Lengths of 200 cashmere fibers were measured with a steel rule, and 400 fiber diams were measured using a microprojector [13]. After the separation of cashmere and wool, the cashmere was washed, dried, and weighed [13], the net cashmere fiber rate (%) = net cashmere fiber weight/fleece weight×100%.

### Statistical analysis

All of the data were analyzed using the analysis of variance (ANOVA) procedure of the Statistical Analysis System (SAS) [24]. The ANOVA used the following model: Y_i_ = u+M_i_+e_i_, where u is the overall mean. M_i_ is the fixed effect of the photoperiod treatments (i = 1,2), and e_i_ is the random residual error. Significant differences between mean values were identified by using Duncan’s test. Significance was defined as p≤0.05.

## RESULTS

### Live weight

As shown in Table 2, although there was no significant difference in the initial live weights of goats between SDPP and NDPP groups in May (p>0.05), the average live weight in the SDPP group was increased by 7.17% in July (p>0.05), 4.01% in September (p>0.05), and 3.76% in October (p>0.05) compared with that of NDPP group.

### Nutrient digestibility

No significant difference in DMI of cashmere goats was observed between SDPP and NDPP groups in July, September, or October (p>0.05). The digestibility of CP in the SDPP group was significantly higher (p<0.05) than in the NDPP group, although there was no significant difference (p>0.05) in the digestibility of DM and NDF between SDPP and NDPP groups in July or October. In addition, the DM, NDF, and CP digestibility of cashmere goats in SDPP group were increased (p<0.05) compared with the NDPP group in September (Table 3).

### Secondary hair follicle activity and melatonin concentration

As shown in Table 4, 5, and Figure 2, the higher SHF activity of the skin and greater MEL concentration in plasma of goat were found in the SDPP group compared to the NDPP group in July (p<0.05), September (p>0.05), and October (p>0.05).

### Fiber length, diam, weight and net fiber rate of goats

Compared to the NDPP group, the cashmere fiber length and weight of goats (p<0.05) were significantly increased in the SDPP group. Moreover, a decreased fiber diam. and increased net fiber rate were observed in the SDPP group (p>0.05) (Table 6).

## DISCUSSION

### Live weight and nutrient digestibility

The results on the live weight and nutrient digestibility indicated that in response to a short daily photoperiod, the live weight and nutrient digestibility of cashmere goats increased rapidly. In the present study, the live weight of cashmere goats in the SDPP group increased by 3.76% under short photoperiodic conditions and the digestibility of DM, CP, and NDF in the SDPP group was greater than in the NDPP group; this was were in agreement with the results of Wang [13] and Li et al [25]. Similarly, Wang et al [14] demonstrated that a short daily photoperiod could be rewarding to increase the deposition of body fat, reduce the deposition of body protein, and accelerate cashmere growth of goats by enhancing nitrogen distribution to cashmere fibers, in effect reprogramming the distribution of body nitrogen and cashmere nitrogen. Exposed to an artificially short photoperiod, metabolic regulation is modified; CP digestibility was especially affected in our animals during the entire experimental period. On one hand, the muscle and cashmere growth of the goats required more protein, so that the rates of protein synthesis and degradation were increased. On the other hand, the nutrients might have increased in digestibility because of increased secretion of MEL by the pineal gland, or it might have been due to reduced activity, weakened intestinal motility, or the extended time of food remaining in the digestive tract.

### Secondary hair follicle activity and melatonin concentration

The rhythmicity of HF cycling from a phase of active fiber production to tissue regression and through rapid resting phases in mammals results from seasonal changes; this provides an important mechanism for regulating the length of hair and allows for the periodic shedding of fur [12,26]. In contrast, the non-modulated fine inner hairs of PHFs and the cashmere produced by SHFs consist of hair from the basement membrane of the skin in cashmere goats [27]. The number and density of SHFs affect the production and diam. of the cashmere fibers and thus determine the value of the cashmere fleece [28]. Furthermore, as a cyclic biological system, the seasonal activity of SHFs controls the growth of cashmere fibers in the skin of cashmere goats [29,30]. In this study, each HF group included three PHFs and each PHF was surrounded by a number of SHFs, which were seen clearly from paraffin sections. The activity of SHFs in short daily photoperiod conditions was higher in the SDPP group in July (p<0.05), September (p>0.05), and October (p>0.05) (Figure 2). Current research indicates that as a critical intermediary, the circulating MEL level plays a crucial role in the mechanism connecting photoperiod and cashmere growth. An increased MEL level can lead to a direct increase in the activity of SHFs and subsequently promote cashmere growth in goats experiencing a short daily photoperiod [11,13–15]. From May to August, the SHFs continue to the anagen phase during which they are gradually formed from the telogen phase at the beginning of a new cashmere growth cycle. Moreover, the SHFs of Inner Mongolia white cashmere goats that had completed the reconstruction by August to September and were displaying the highest activity level were at the procatagen phase in October [31–33]. Furthermore, our study inferred that the SHF activity of cashmere goats was significantly increased in the SDPP group; this was attributed to the pineal gland secreting more MEL under short daily photoperiod conditions in July. The activity of SHFs continued to increase until September and October, which might suggest that the anagen phase of SHFs and the rapid growth period of cashmere were prolonged by the short daily photoperiod conditions.

### Cashmere growth performance

The quality of cashmere mainly depends on the diam., length and production of cashmere fibers in the goats, and this is closely linked to economic returns. As the most luxurious fiber material in the textile industry, cashmere fibers less than 35 mm in length will lose value, and fibers with a diam. more than 16.5 μm reduce the quality of the cashmere. In previous studies, not only the cashmere fiber length and density of Shanbei cashmere goats were increased, but also the cashmere fiber weight of Northwest Tibet cashmere goats was higher under short daily photoperiod conditions [34,35]. In the current study, the longer cashmere fiber length (p<0.05), higher fiber weight (p<0.05), and finer cashmere diam. (p>0.05) were observed under short daily photoperiod conditions, and this was in agreement with the results of Xu [36] and Lin [21].

Previous results showed that nitrogenous compounds were partitioned more to cashmere growth in goats in the SDPP group than in the NDPP group [13,14]. As we all know that cashmere growth requires more protein, and the higher CP digestibility may have been a key factor for promoting cashmere growth in the SDPP group. Constant MEL availability during the short daily photoperiod may have accelerated SHF activity to induce cashmere growth.

## CONCLUSION

In summary, the current study demonstrated that DM digestibility in September, NDF digestibility in September, CP digestibility during entire experimental period, SHF activity in July, MEL concentration in July, cashmere fiber length in October, and fiber weight of goats in October were significantly increased under short daily photoperiod conditions. The cashmere production of Inner Mongolia white cashmere goats in the SDPP group was increased without adverse effects on the diam. of cashmere fibers (ME, 8.34 MJ/kg; CP, 11.16%; short daily photoperiod: 7 light:17 dark).

## Figures and Tables

**Table 1:** The photoperiod and ingredients in the diet1)

Ingredient2)	Level (%)	Nutrients3)	Concentration
	
Alfalfa hay	35.00	ME (MJ/kg)	8.34
Corn stalk	25.00	DM (%)	91.38
Corn grain	15.00	CP (%)	11.16
Concentrated feed	25.00	NDF (%)	46.56
Concentrate to forage ratio	40:60	NFC (%)	33.70
		NFC/NDF	0.72
		Ca (%)	0.80
		P (%)	0.36
		Ca/P	2.22/1
		ERDP/UDP	1.65

ME, metabolizable energy; DM, dry matter; CP, crude protein; NDF, neutral detergent fiber; NFC, non-fibrous carbohydrates; Ca, calcium; P, phosphorus; ERDP, effective ruminally degradable protein; UDP, undegradable protein.

1) Diet for both period was the same. Natural daily photoperiod group (NDPP group: 10 to 16 h light, n = 12), short daily photoperiod group (SDPP group: 7 h light:17 h dark, n = 12).

2) Ingredients in the diet is dry matter basis.

3) Determined by the laboratory analyses.

**Table 2:** Effects of photoperiod on mean live weight of goats

Month	NDPP group1)	SDPP group1)	SEM	p-value
May	20.90	21.08	0.847	0.881
July	22.58	24.20	0.629	0.099
September	26.68	27.75	0.360	0.062
October	28.19	29.25	0.502	0.166

SEM, standard error of the mean.

1) Natural daily photoperiod group (NDPP group: 10 to 16 h Light, n = 12), short daily photoperiod group (SDPP group: 7 h light:17 h dark, n = 12).

**Table 3:** Effects of photoperiod on mean nutrient digestibility1) of goats

Items	NDPP group2)	SDPP group2)	SEM	p-value
DMI in July (kg) (July)	0.83^a^	0.86^a^	0.025	0.416
DM digestibility (%)	73.74^a^	75.78^a^	2.384	0.567
NDF digestibility (%)	68.56^a^	70.01^a^	1.820	0.593
CP digestibility (%)	69.80^b^	74.81^a^	1.114	0.019
DMI in September (kg) (September)	0.97^a^	0.98^a^	0.027	0.734
DM digestibility (%)	71.69^b^	79.45^a^	2.217	0.048
NDF digestibility (%)	62.56^b^	70.45^a^	2.123	0.039
CP digestibility (%)	68.11^b^	75.98^a^	1.886	0.026
DMI in October (kg) (October)	1.01^a^	1.04^a^	0.024	0.507
DM digestibility (%)	77.25^a^	78.83^a^	2.729	0.696
NDF digestibility (%)	65.82^a^	68.82^a^	2.810	0.478
CP digestibility (%)	68.58^b^	75.86^a^	1.485	0.013

SEM, standard error of the mean; DMI, dry matter intake; DM, dry matter; NDF, neutral detergent fiber; CP, crude protein.

1) Apparent digestibility =100–{feces weight×nutrient content in feces/dry matter intake×nutrient content in the diet (%)} [23].

2) Natural daily photoperiod group (NDPP group: 10 to 16 h light, n = 12), short daily photoperiod group (SDPP group: 7 h light:17 h dark, n = 12).

a,b Within a row, means without a common letters differ (p<0.05).

**Table 4:** Effects of photoperiod on mean secondary hair follicle activity1) of goats

Month	NDPP group2)	SDPP group2)	SEM	p-value
July (%)	69.52^b^	78.88^a^	2.260	0.015
September (%)	79.48^a^	80.04^a^	2.130	0.855
October (%)	74.60^a^	80.73^a^	2.094	0.065

SEM, standard error of the mean.

1) Secondary hair follicle activity = active secondary hair follicles/total secondary hair follicles×100%

2) Natural daily photoperiod group (NDPP group: 10 to 16 h light, n = 12), short daily photoperiod group (SDPP group: 7 h light:17 h dark, n = 12).

a,b Within a row, means without a common letters differ (p<0.05).

**Table 5:** Effects of photoperiod on mean melatonin concentration in plasma of goats

Month	NDPP group1)	SDPP group1)	SEM	p-value
July (ng/L)	47.03^b^	62.05^a^	3.487	0.023
September (ng/L)	44.42^a^	47.36^a^	5.798	0.732
October (ng/L)	49.54^a^	56.63^a^	4.269	0.285

SEM, standard error of the mean.

1) Natural daily photoperiod group (NDPP group: 10 to 16 h light, n = 12), short daily photoperiod group (SDPP group: 7 h light:17 h dark, n = 12).

a,b Within a row, means without a common letters differ (p<0.05).

**Table 6:** Effects of photoperiod on mean cashmere fiber length1), diameter2), weight3) and net fiber rate4) of goats at the end of the experiment (in October)

Items	NDPP group5)	SDPP group5)	SEM	p-value
Fiber length (cm)	6.76^b^	8.64^a^	0.442	0.013
Fiber diameter (μm)	15.38^a^	15.11^a^	0.183	0.317
Fiber weight (g)	3.04^b^	3.35^a^	0.090	0.033
Net fiber rate (%)	45.98^a^	46.39^a^	2.802	0.924

SEM, standard error of the mean.

1) Lengths of 200 cashmere fibers were measured with a steel rule.

2) Diameters 400 of cashmere fibers were measured using a microprojector [13].

3) Cashmere and wool samples from behind the right shoulder blade were dyed on May 15 and were shorn near to the skin on October 15. Both dyeing and shearing samples measured 10×10 cm. After the separation of cashmere and wool, the cashmere was washed, dried, and weighed [13].

4) Net cashmere fiber rate (%) = net cashmere fiber weight/fleece weight×100%.

5) Natural daily photoperiod group (NDPP group: 10 to 16 h light, n = 12), short daily photoperiod group (SDPP group: 7 h light:17 h dark, n = 12).

a,b Within a row, means without a common letters differ (p<0.05).
